# Impact of COVID-19 on the Care of Patients with HIV Infection

**DOI:** 10.3390/jcm12123882

**Published:** 2023-06-07

**Authors:** Marta Rosas Cancio-Suárez, Cecilia Alonso, María Jesús Vivancos, María Jesús Pérez-Elías, María José Cárdenas, Manuel Vélez-Díaz-Pallarés, María Dolores Corbacho, Laura Martín-Pedraza, Alfonso Muriel, Javier Martínez-Sanz, Santiago Moreno

**Affiliations:** 1 Department of Infectious Diseases, Hospital Universitario Ramón y Cajal, Instituto Ramón y Cajal de Investigación Sanitaria IRYCIS, Carretera de Colmenar Km 9.1, 28034 Madrid, Spain; 2 CIBERINFEC, 28029 Madrid, Spain; 3 Microbiology Department, Hospital Universitario Ramón y Cajal, Instituto Ramón y Cajal de Investigación Sanitaria IRYCIS, Carretera de Colmenar Km 9.1, 28034 Madrid, Spain; 4 Pharmacy Department, Hospital Universitario Ramón y Cajal, Instituto Ramón y Cajal de Investigación Sanitaria IRYCIS, Carretera de Colmenar Km 9.1, 28034 Madrid, Spain; 5 Biostatistics Department, Hospital Universitario Ramón y Cajal, Instituto Ramón y Cajal de Investigación Sanitaria IRYCIS, 28034 Madrid, Spain; 6 Department of Medicine, University of Alcalá de Henares, Guadalajara Campus, 28801 Alcalá de Henares, Spain

**Keywords:** SARS-CoV-2, COVID-19 pandemic, HIV, PLWH, holistic care

## Abstract

The COVID-19 pandemic and associated lockdown measures have been associated with substantial disruptions to health care services, including screening for human immunodeficiency virus (HIV) and management of people living with HIV (PLWH). Data from 3265 patients were examined in a retrospective cohort study. We compared outpatient follow-up for PLWH, the number of new patients, treatment adherence, hospitalizations, and deaths during the “pandemic period” (March 2020 to February 2021), the “pre-pandemic period” (the equivalent time frame in 2019), and the “post-pandemic period” (March to September 2021). During the pandemic period, the number of new patients seen at the HIV clinic (116) as well as the requested viral load tests (2414) decreased significantly compared to the pre-pandemic (204 and 2831, respectively) and post-pandemic periods (146 and 2640, respectively) (*p* < 0.01 for all the comparisons). However, across the three study periods, the number of drug refills (1385, 1330, and 1411, respectively), the number of patients with undetectable viral loads (85%, 90%, and 93%, respectively), and the number of hospital admissions among PLWH remained constant. Despite the COVID-19 pandemic’s impact, our findings show stability in the retention of clinical care, adherence to treatment, and viral suppression of PLWH, with no significant impact on hospitalization rates or all-cause mortality.

## 1. Introduction

The COVID-19 pandemic has significantly impacted many aspects of our lives, including healthcare. During the COVID-19 pandemic, many countries implemented lockdowns and other public health measures to limit the spread of the virus. These measures often resulted in the cancellation or postponement of non-urgent medical appointments and procedures [1].

On 14 March, Royal Decree 463/2020 was published in Spain, declaring a state of alarm to manage the global health crisis. It instituted the lockdown and confinement of all Spaniards, with the exception of those engaging in essential activities [2,3]. The fear of the unknown, the panic associated with contagion risk, the overloaded emergency departments and hospitals, and the redistribution of health staff to manage the public health crisis are factors that contributed to the diminished care available to patients with chronic diseases. Since patients were formally instructed to stay at home due to the high transmissibility of SARS-CoV-2, most non-urgent consultations, procedures, and follow-up or routine appointments were cancelled or postponed [3,4].

The impact of COVID-19 on long-term health outcomes for patients with chronic conditions is characterized as a “second hit” [5], and will likely disproportionately affect vulnerable populations. One such group is people living with HIV (PLWH). As HIV infection is already a chronic disease whose follow-up requires frequent visits to the hospital (e.g., consultations, tests, pharmacy, visits to other services), it was foreseeable that the isolation and confinement measures adopted would have had an impact on the care of PLWH [6,7,8,9].

In addition to the logistical challenges of accessing care during a pandemic, PLWH may also face increased psychological stress and anxiety due to the heightened risk of COVID-19 complications for those with underlying health conditions [10]. This stress can further impact adherence to treatment and overall health outcomes [10].

Despite the considerable challenges posed by the COVID-19 pandemic, healthcare providers and organizations have developed various strategies to mitigate the impact of COVID-19 on HIV care. Our service has implemented several measures to ensure continuity of care for people living with HIV. We have utilized telemedicine and virtual appointments to enable patients to assess their clinical statuses and receive medical advice without the need for physical hospital visits. Additionally, we have provided telephone-based communication of laboratory test results, such as viral load and CD4 counts. We have also placed increased emphasis on home-based care and self-management. To ensure uninterrupted care for newly diagnosed patients, we have maintained an open in-person consultation service. Furthermore, we have made adjustments to the dispensing of medication. In Spain, antiretroviral medication is usually dispensed in hospitals, requiring patients to visit the pharmacy service in person. As a result, we modified our medication-dispensing procedures by collaborating with the Pharmacy Service to devise a contingency plan involving home delivery of antiretroviral medication to patients, thus reducing the risk of COVID-19 exposure.

The COVID-19 pandemic has had a profound impact on healthcare systems and the provision of care for people living with HIV. However, it has also highlighted the importance of addressing health disparities and social determinants of health, which may disproportionately affect vulnerable populations, including PLWH. While the pandemic has presented numerous challenges, healthcare providers and organizations have demonstrated resilience and innovation in adapting to these challenges and ensuring that PLWH continue to receive the care they need.

The aim of this study was to examine the impacts of the COVID-19 pandemic on the care of PLWH, with a specific focus on minimizing loss to follow-up. We compared treatment adherence; control of immunovirological status; and the number of hospital admissions, new enrollments, and deaths during the pandemic with those over two periods of similar duration (the “pre-pandemic” period and “post-pandemic” periods, respectively).

This study also provides valuable insights into the impacts of the COVID-19 pandemic on the care of PLWH and the measures taken to mitigate those impacts. Moving forward, it is essential to continue prioritizing the care of PLWH and addressing the underlying social determinants of health in order to improve health outcomes for vulnerable populations. We hope that this study can contribute to improving the ongoing care of PLWH. We will need to redouble our efforts to achieve the newly proposed global 95–95–95 targets set by UNAIDS, which aim to ensure that 95% of people diagnosed with HIV are being treated and have a suppressed viral load.

## 2. Materials and Methods

We conducted a retrospective cohort study in the Infectious Diseases Department of the Ramon y Cajal University Hospital in Madrid, Spain. The study population consisted of 3265 individuals diagnosed with HIV infection and registered in the Patient Information and Management Systems Service of Ramón y Cajal University Hospital. The data were analyzed during the period from March 2019 to September 2021.

For the purpose of this study, we divided this period of time into three distinct periods spanning up to 12 months: the pre-pandemic period, from March 2019 to February 2020; the pandemic period, from March 2020 to February 2021; and the post-pandemic period, from March 2021 to September 2021, prior to the availability of vaccinations.

To analyze the impact of the pandemic on HIV care, we examined the following indicators: (1) the number of PLWH that first visited the clinic; (2) treatment adherence, as measured by the frequency with which the patients picked up the medication at the hospital pharmacy; (3) detectability of viral load; and (4) hospitalizations and deaths. To obtain these data, we collaborated with the Information Systems and Patient Management Service; the Microbiology Department, which provided information on viral loads; and the Pharmacy Department, which provided information on antiretroviral drugs dispensed during these periods.

We compared these five variables across different periods. Firstly, we compared the variables between the pre-pandemic and pandemic periods, and then the months of the pandemic with those of the post-pandemic period. Thus, we reviewed whether the number of first visits increased or decreased, and, consequently, the number of HIV diagnoses during these periods. We reviewed the supply of antiretroviral drugs by the Pharmacy Service of Ramón y Cajal University Hospital, since patients went from having to collect their medications from the hospital every three months to being able to receive them at home thanks to Resolution 197/2020 of the General Directorate of Economic and Financial Management and Pharmacy of the Community of Madrid, published on 31 March 2020, which authorized home medication delivery. We also analyzed whether undetectable viral loads increased or decreased during the pandemic due to this change in drug administration and medical consultations. Finally, we compared the number of hospitalizations and deaths during these periods, differentiating between those caused by SARS-CoV-2 and other causes unrelated to it.

The data were expressed as a mean ± the standard deviation (SD) or as a proportion (%). Categorical variables were expressed as absolute frequencies and percentages. We compared continuous variables using a Student’s t-test or a Mann–Whitney U test, according to their distribution. The associations between categorical variables were assessed using a chi-squared test or a Fisher’s exact test, as appropriate. A logistic regression model, weighted by the duration of each period, was used to analyze binary categorical variables such as first visits, undetectable viral loads, hospitalizations, and deaths, with estimated 95% confidence intervals. A regression model weighted by the number of months per period was employed for the analysis of continuous variables, such as the number of viral load tests performed and the number of first visits. Statistical significance was identified at *p* < 0.05. Statistical analyses were performed using Stata (StataCorp. 2019. Stata: Release 16.1. Statistical Software; StataCorp LLC, College Station, TX, USA).

According to current regulations, since this was an observational study without interventions on patients, and it was retrospective in nature, obtaining informed consent was not required. However, the study protocol was submitted to the ethics committee of Ramón y Cajal Hospital, and after their analysis, they approved the study. The approval from the Ethics Committee was obtained on 16 October 2021.

## 3. Results

Within the study period, 3265 patients with HIV were examined at our clinic, resulting in 8860 outpatient visits, including 466 first visits (5.26%). We observed a decrease in the number of new PLWH visiting the clinic from the beginning of the pandemic period, which included only 116 out of 2805 visits (3.97%). This percentage was significantly lower compared to the pre-pandemic period (204 visits, 6.71%, *p* < 0.05) and the post-pandemic period (146 visits, 5.25%, *p* < 0.05) (Table 1).

Similarly, we observed that the proportion of initial visits was 73.87% higher in the pre-pandemic period compared to the pandemic period (OR = 1.74 CI95% 1.76–2.20 *p* < 0.05). In the post-pandemic period, the number of initial visits was 28.28% higher than in the pandemic period (OR = 1.28 CI95% 0.10–1.65 *p* = 0.05).

**Figure 1:**
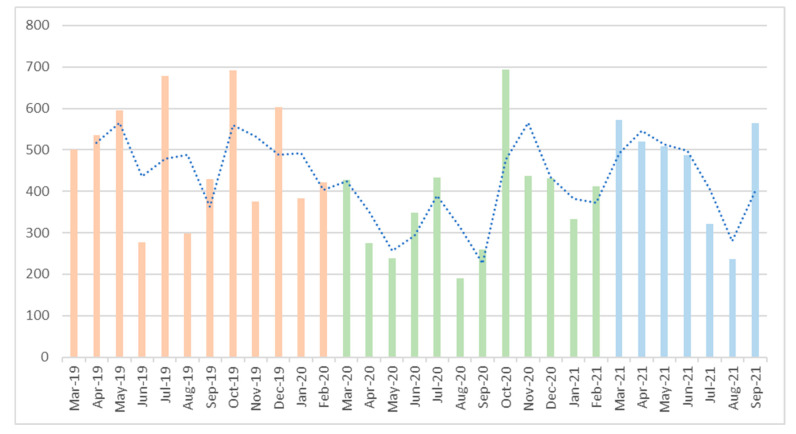
Total viral loads requested.

Throughout the longest stretch of time that comprised the pandemic period (March 2020 to February 2021), the total number of viral load tests performed decreased compared to the number of tests performed in the pre-pandemic and the post-pandemic periods (Figure 1). During the pandemic period, 2414 viral loads were analyzed, compared to 2831 and 2640 analyzed in the pre- and post-pandemic periods, respectively (both *p* < 0.05). Linear regression results showed that the average number of viral load tests performed during the pandemic was 1.47 per patient. In the pre-pandemic period, more viral load tests were performed—specifically, 0.47 more tests on average than during the pandemic period (*p* < 0.05). In the post-pandemic period, the number of viral load tests performed decreased slightly compared to the pandemic period; it decreased by 0.03 (*p* < 0.05).

Although the number of plasma viral load tests decreased during the pandemic period, the proportion of undetectable viral loads (referring to patients who had viral suppression with a viral load less than 50 copies/mL) [11] remained stable. Specifically, throughout the pandemic period, 2182 out of 2560 viral load tests analyzed showed viral suppression, corresponding to a percentage of 90.39%. This percentage was comparable to the pre-pandemic period, where 2408 out of 2831 tests (85.06%) showed viral suppression, as well as the post-pandemic period, where 2496 out of 2696 tests (92.65%) showed viral suppression (Table 1). These differences were statistically significant.

In fact, the proportion of patients with at least one detectable viral load during the pre-pandemic period was 65.21% higher compared to the pandemic period (OR = 1.65 CI95% 1.39–1.96 *p* < 0.05). In the post-pandemic period, the proportion of patients with at least one detectable viral load was reduced by 25.40% compared to that measured during the pandemic period (OR = 0.75 CI95% 0.61–0.91 *p* < 0.05).

**Figure 2:**
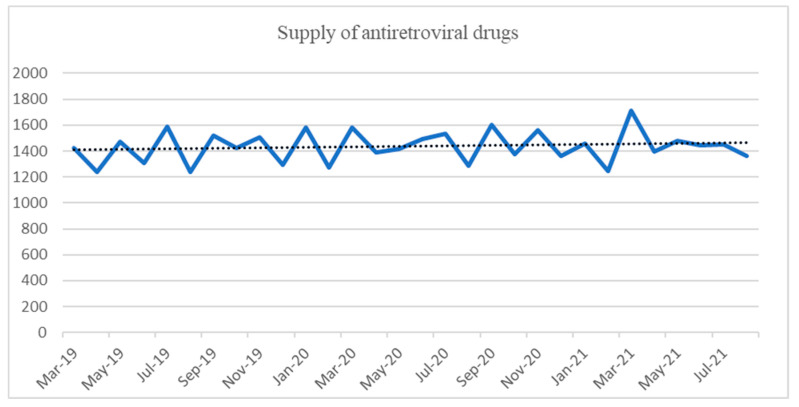
Evolution of the supply of antiretroviral drugs according to the moment of the pandemic.

We did not observe any change in adherence to treatment, which was determined using pharmacy records (Figure 2). The median number of drug refills during the pandemic period was 1454, compared to 1425 (*p* = 0.39) and 1449 (*p* = 0.84) during the pre- and post-pandemic periods, respectively.

We observed no impact of the pandemic on the number of hospitalizations among PLWH for reasons other than COVID-19 in our hospital. The total number of monthly admissions remained stable across the three periods. Notably, in-hospital mortality rates (in terms of odds) of PLWH during the pandemic period was reduced compared to the pre-pandemic period (OR 2.29, CI 1.49–3.53, *p* < 0.001). In other words, the risk of death, in terms of odds, was approximately twice as high in the pre-pandemic period compared to the pandemic period. The differences found between the pandemic and post-pandemic periods were not significant (OR 1.11, CI 0.67–1.82, *p* = 0.68). None of the recorded deaths among PLWH were associated with a SARS-CoV-2 infection, and most deaths were related to non-AIDS events, especially neoplasms (accounting for 38.4% of all deaths). Only 7.7% of deaths across the three periods were related to HIV infection (three lymphomas, one Kaposi’s sarcoma).

Overall, our study highlights the impact of the COVID-19 pandemic on the healthcare of patients with HIV, as evidenced by the significant decrease in clinic visits during the pandemic period. Despite this decrease, we did not observe any adverse effects on the proportion of patients with undetectable viral loads or adherence to ART. Furthermore, our findings suggest that the pandemic may have had a protective effect on the in-hospital mortality rates of patients with HIV.

## 4. Discussion

Our study examined the effect of COVID-19 restrictions on the quality of HIV care at our hospital. We showed that, although the number of viral load tests decreased throughout the months when public health measures were most stringent, the proportion of patients with undetectable viral loads remained stable. Behavioral modifications among individuals living with HIV (PLWH), including adherence to medication regimens, engagement in care, and adoption of preventive measures, play a critical role in achieving and sustaining viral suppression. While our study did not directly measure behavioral changes, we acknowledge their importance within the realm of HIV care and treatment.

We also found that the number of patients that continued to use their medications, the number of hospitalizations, and the number of deaths were not negatively influenced by lockdown restrictions or other measures to mitigate the impact and spread of SARS-CoV-2. Similar findings were widely reported from various countries (Spain, USA, Perú, Uganda, etc.) [12,13,14,15]. This study provides evidence that the strategies implemented during the COVID-19 pandemic effectively minimized the potential impact on individuals living with chronic conditions such as HIV infection. These findings underscore the critical role of specific healthcare practices and indicates that certain hospital practices may have allowed patients to continue to receive care when the overall healthcare system had been severely disrupted.

To mitigate the impacts of the pandemic on the care available to PLWH, it is imperative to rely on the HIV care continuum (HCC) [16] as a guide. The HCC was designed to model progressive stages of HIV care. This continuum consists of five steps: (i) diagnosis; (ii) linkage to care; (iii) retention in care; (iv) adherence to antiretroviral therapy; and (v) viral suppression. Due to quarantine measures and fewer in-person hospital visits, there have been reports of reduced access to routine HIV testing, linkage to care, and retention in care [7,16].

Although our “first visit” clinic remained open to enable care for people with new diagnoses, various community organizations and associations that connect patients with hospital centers reduced their field activities due to lockdown measures and social distancing orders. One option to maintain access to testing included distributing self-diagnostic tests in pharmacies for people at a higher risk of contracting HIV, a practice carried out in Ottawa (Canada) [17].

For patients who were already aware of their HIV status, COVID-19 lockdown measures may have limited their adherence to antiretroviral therapy and viral suppression. The integration of an ART dispensing program, coordinated by the hospital pharmacy, was implemented during the most stringent public health lockdown measures. This new initiative helped to ease concerns that ART may have been interrupted due to difficulties traveling to the hospital and for safety reasons, and allowed patients to avoid a crowded hospital pharmacy. The present study examined our experience managing patient needs and successfully maintained the administration of ART throughout the pandemic, where no differences were observed between the pre-pandemic and pandemic stages.

Various studies have described the experience in other centers regarding the management of people living with HIV (PLWH) during the pandemic [17,18]. The COVID-19 pandemic was a situation that compelled us to surpass ourselves, both from a clinical perspective and from the patient’s standpoint. Telemedicine was one of the most widely utilized resources, as well as outpatient dispensation of antiretroviral therapy (ART) [19]. However, few studies have reported data on changes in viral suppression rates when comparing time periods during the pandemic with pre-pandemic periods [19]. In our study, we describe the data on changes in viral suppression rates. These data, along with changes in healthcare delivery and challenges in accessing ART, will be important for fully understanding the long-term health impacts of the pandemic on PLWH.

Overall, our findings suggest that patients with HIV are currently achieving better viral suppression despite the challenges posed by the COVID-19 pandemic. The availability of more effective ART regimens, increased awareness of the importance of adherence, and the implementation of the HCC model may all contribute to these positive outcomes. Continued efforts to maintain access to care and support consistent adherence to ART will be critical for sustaining these gains and improving patient outcomes over the long term.

These practices made it easier for our patients to continue with consistent therapeutic compliance. Home dispensing of ART can similarly support patient access to care and reduce the burden on the hospital system [20,21].

Future research endeavors should focus on investigating and quantifying specific behavioral changes among PLWH and their correlation with viral suppression outcomes. This could entail assessing patterns of medication adherence, examining healthcare utilization trends, and exploring shifts in risk behaviors and preventive practices.

By gaining a comprehensive understanding of the behavioral aspects and psychosocial factors that impact viral suppression, healthcare providers and policymakers can tailor interventions to address the unique challenges faced by PLWH. Moreover, fostering a patient-centered approach that encourages shared decision making and provides continuous support may further enhance the long-term maintenance of viral suppression.

Regarding the number of hospitalizations and deaths among PLWH, we did not observe a greater number of hospitalizations or deaths due to the SARS-CoV-2 infection across the general population, as reported in other research [22,23]. However, despite efforts to maintain care for HIV patients, the number of deaths throughout the pandemic and post-pandemic period is increasing. Most recorded deaths are associated with secondary or non-AIDS events. This observation may be due to the unchanged rate of undetectable persons across these time periods.

In summary, people with chronic illnesses such as HIV rely heavily on regular consultations with their healthcare providers to monitor their disease and/or risk factors associated with their disease [24]. As described above, the pandemic has impacted the rate of disease diagnosis, and it has negatively impacted the quality of care for patients with chronic diseases [25,26]. In the hospital examined in this study, we adopted some measures that successfully mitigated the impact of the pandemic on patient care that may be adopted to further support consistent patient care. These measures include maintaining access to a “first visit” clinic to facilitate the continuity of care, home delivery of ART, and rapid initiation of ART. The ART-dispensing practices contributed to HIV treatment adherence and decreased the risk of COVID-19 transmission. Although our findings demonstrated consistency in the retention and viral suppression of PLWH, careful monitoring over the coming years is critical. Core healthcare priorities must be sustained, and healthcare decision makers need to creatively adapt patient care practices to manage shifting public health challenges.

## Figures and Tables

**Table 1:** Time frame comparison. Data are n (%), median, or *p* value.

	Pandemic	Pre-Pandemic	*p* Value	Pandemic	Post-Pandemic	*p* Value
First consultation	116 (3.97%)	204 (6.71%)	<0.05	166 (3.97%)	146 (5.25%)	0.05
VL performed	2414	2831	<0.05	2414	2640	<0.05
Undetectable VL	2182 (90.39%)	2408 (85.06%)	<0.05	2182 (90.39%)	2446 (92.65%)	<0.05
Median drug refills	1454	1425	0.395	1454	1449	0.843

## Data Availability

The data presented in this study are available upon request from the corresponding author.
